# Understanding the Sensory Loss–Cognitive Function Relationship in Older Adults: Biomarker or Causal Risk Factor?

**DOI:** 10.1093/geroni/igaa057.2920

**Published:** 2020-12-16

**Authors:** Jennifer Deal, Heather Whitson

## Abstract

Sensory impairment in older adults is common, over 55% of Americans 60 years and older have either hearing or vision impairment, and it is linked to accelerated cognitive decline and increased risk of incident dementia in population-based observational studies. However, whether sensory impairment is a marker or a cause of cognitive decline and dementia is unknown. Both sensory impairment and cognitive decline/dementia may be caused by a common underlying pathology (e.g., microvascular disease), or sensory impairment may be a marker of dementia-related pathological changes in the brain. Alternatively, causal mechanisms include increased cognitive load, changes brain structure/function, depression, social isolation and/or reduced activity. This session will investigate the role of sensory impairment in cognitive decline and dementia in older adults and discuss the ramifications of these different possibilities for risk prediction and stratification, and potentially, for disease prevention. The co-occurrence of multiple sensory deficits will be described, and the potential utility of the use of retinal signs as predictive markers for cognitive decline/dementia will be discussed. We will also describe current evidence for both non-causal and causal relationships between sensory impairment and cognition with a focus on hearing impairment. Finally, we will describe the relationship of dual sensory (both hearing and vision) impairment on cognitive performance and dementia in a biracial population-based study.

